# C_60_ fullerene as promising therapeutic agent for correcting and preventing skeletal muscle fatigue

**DOI:** 10.1186/s12951-016-0246-1

**Published:** 2017-01-13

**Authors:** Yurij I. Prylutskyy, Inna V. Vereshchaka, Andriy V. Maznychenko, Nataliya V. Bulgakova, Olga O. Gonchar, Olena A. Kyzyma, Uwe Ritter, Peter Scharff, Tomasz Tomiak, Dmytro M. Nozdrenko, Iryna V. Mishchenko, Alexander I. Kostyukov

**Affiliations:** 1 Department of Biophysics, Taras Shevchenko National University of Kyiv, Volodymyrska Str. 60, Kiev, 01601 Ukraine; 2 Department of Movement Physiology, Bogomoletz Institute of Physiology, Bogomoletz Str. 4, Kiev, 01024 Ukraine; 3 Department of Hypoxic States Investigation, Bogomoletz Institute of Physiology, Bogomoletz Str. 4, Kiev, 01024 Ukraine; 4 Joint Institute for Nuclear Research, Joliot-Curie Str. 6, Dubna, Moscow Region Russia; 5 Institute of Chemistry and Biotechnology, Technical University of Ilmenau, Weimarer Str. 25, 98693 Ilmenau, Germany; 6 University of Physical Education and Sport, Kazimierza Górskiego Str.1, 80-336 Gdansk, Poland; 7 Lesia Ukrainka Eastern European National University, Volya Avenue 13, Lutsk, 43025 Ukraine

**Keywords:** C_60_ fullerene, Skeletal muscles fatigue, Electrical stimulation, Oxidative stress markers, Antioxidant system

## Abstract

**Background:**

Bioactive soluble carbon nanostructures, such as the C_60_ fullerene can bond with up to six electrons, thus serving by a powerful scavenger of reactive oxygen species similarly to many natural antioxidants, widely used to decrease the muscle fatigue effects. The aim of the study is to define action of the pristine C_60_ fullerene aqueous colloid solution (C_60_FAS), on the post-fatigue recovering of *m. triceps surae* in anaesthetized rats.

**Results:**

During fatigue development, we observed decrease in the muscle effort level before C_60_FAS administration. After the application of C_60_FAS, a slower effort decrease, followed by the prolonged retention of a certain level, was recorded. An analysis of the metabolic process changes accompanying muscle fatigue showed an increase in the oxidative stress markers *H*_*2*_ *O*_*2*_ (hydrogen peroxide) and *TBARS* (thiobarbituric acid reactive substances) in relation to the intact muscles. After C_60_FAS administration, the *TBARS* content and *H*_*2*_ *O*_*2*_ level were decreased. The endogenous antioxidant system demonstrated a similar effect because the *GSH* (reduced glutathione) in the muscles and the *CAT* (catalase) enzyme activity were increased during fatigue.

**Conclusions:**

C_60_FAS leads to reduction in the recovery time of the muscle contraction force and to increase in the time of active muscle functioning before appearance of steady fatigue effects. Therefore, it is possible that C_60_FAS affects the prooxidant-antioxidant muscle tissue homeostasis, subsequently increasing muscle endurance.

## Background

Skeletal muscle fatigue is the defence mechanism against overload and leads to the development of painful muscle sensitivity [1–3]. Muscle fatigue develops after physical activities of varying intensities and often leads to acute pain, which can then lead to various chronic disease states [4, 5]. Muscle fatigue is a result of the products of incomplete oxygen oxidation, such as reactive oxygen species (ROS), including peroxides, free radicals, and oxygen ions [6]. During the course of lipid peroxidation, unsaturated fatty acids are formed from various fatty acid derivatives and metabolites, such as malondialdehyde and hydroperoxide fatty acid [7]. The excessive accumulation of ROS (oxidative stress) can lead to significant functional changes due to damage to different cell components [8]. An example is the lipid peroxidation of biological membranes, which promotes the disruption of their structure and increases their permeability [9]. Cell protection against such damage is provided by the antioxidant system. Mach et al. [10] used pycnogenol as an antioxidant, and its use is accompanied by an increase in the levels of both oxidized and reduced NAD^+^ in the serum, as well as increased muscle strength. In studies of muscle fatigue, endogenous antioxidants, such as an N-acetylcysteine [11] and β-alanine [12], are widely used and speed up the muscle recovery process after fatigue. In this context, bioactive soluble carbon nanostructures, such as the pristine C_60_ fullerenes, may be considered potential antioxidants [13]. C_60_ fullerene easily bonds with up to six electrons, can serve as a powerful scavenger of ROS [13–15], and is superior to the majority of natural antioxidants, including vitamins C and E and carotenoids, in regard to its antioxidant capacity. As a result, it prevents oxidative stress dissemination in thymocytes [16] and shows a protective effect following the ischemia–reperfusion injury of skeletal muscle [17]. Additionally, water-soluble pristine C_60_ fullerenes can penetrate through the plasma membrane of cells [18, 19]. Therefore, the use of C_60_ fullerenes may have a powerful antioxidant effect on the contractile apparatus of striated muscle, thereby facilitating its functional recovery after experimentally induced fatigue.

The aim of this study was to investigate the effect of water-soluble pristine C_60_ fullerenes on the recovery dynamics of the contractile properties of rat *m. triceps surae *(*TS*) after the development muscle fatigue under conditions of long-term activation.

## Methods

### Material preparation and characterization

A highly stable reproducible pristine C_60_ fullerene aqueous colloid solution (C_60_FAS) at a concentration of 0.15 mg/ml was prepared according to a previous protocol [20, 21]. Briefly, for the preparation of C_60_FAS we used a saturated solution of pure C_60_ fullerene (purity >99.99%) in toluene with a C_60_ molecule concentration corresponding to maximum solubility near 2.9 mg/ml, and the same amount of distilled water in an open beaker. The two phases formed were treated in ultrasonic bath. The procedure was continued until the toluene had completely evaporated and the water phase became yellow colored. Filtration of the aqueous solution allowed to separate the product from undissolved C_60_ fullerenes. The pore size of the filter during the filtration of the aqueous solution was smaller than 2 µm (Typ Whatmann 602 h1/2). The purity of prepared C_60_FAS (i.e., the presence/absence of any residual impurities, for example carbon black, toluene phase) was determined by HPLC and GC/MS analysis. The maximal concentration of C_60_ fullerenes in water 0.15 mg/ml was obtained by this method.

**Fig. 1:**
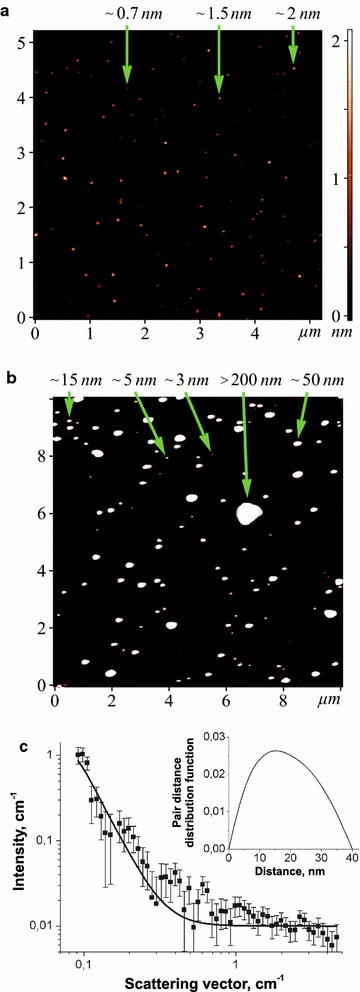
AFM images (tapping mode) of C_60_ fullerene particles on the mica surface, which were precipitated from C_60_FAS with an initial concentration of 0.15 mg/ml (**a**, **b**). *Arrows* indicate the height of the individual particles. Experimental SANS curve (*points*) for C_60_FAS (0.15 mg/ml). Solid lines correspond to the model curve obtained by the IFT procedure. Insert: the pair distance distribution function as a result of the IFT procedure for scattering from C_60_ fullerene nanoparticles present in the C_60_FAS (**c**)

The state of C_60_ fullerenes in aqueous solution was monitored using atomic force microscopy (AFM). Under AFM analysis, the sample was deposited onto a cleaved mica substrate (V-1 Grade, SPI Supplies) by precipitation from an aqueous solution droplet. Sample visualization was performed in semi-contact (tapping) mode (Fig. 1a, b). AFM measurements were performed after the complete evaporation of the solvent.

Small-angle neutron scattering (SANS) measurements (Fig. 1c) were carried out at the YuMO small-angle diffractometer at the IBR-2 pulsed reactor (JINR, Dubna, Russia) in the time-of-flight mode with the two-detector setup [22]. Treatment of the raw data was performed by the SAS program [23].

### Procedure and experimental groups

Male Wistar rats, weighing 280–350 g, were used in the study. The use of the animals was approved by the Ethics Committee of the Institute and performed in accordance with the European Communities Council Directive of 24 November 1986 (86/609/EEC).

The animals were divided into 4 groups. In the experiments, the *m*. *triceps surae* fatigue was induced by electrical stimulation of *n. tibialis.* Saline solution (group 1, n = 6) or C_60_FAS (F-injection) 0.1–0.15 mg/kg (group 2, n = 6) was administered into the left *TS* after the development of fatigue. Then, fatigue of the right *TS* was induced. The data obtained from the ipsilateral (left) side were considered to be the control values vs. those obtained from the contralateral side. The dose range of 0.1–0.15 mg/kg C_60_FAS does not present any acute or subacute toxicity in rats [13]. The rats of group 3 (n = 6; animals with fatigue of both *TS* without any injections) and group 4 (n = 6; intact animals) were used only for biochemical studies. After the experiment, the *TS* of all animals in all groups were removed for biochemical analysis.

It is important to note that a dose of 0.1–0.15 mg/kg C_60_FAS applied in our experiments does not present any acute or subacute toxicity in animals: it was significantly lower than the maximum tolerated dose of C_60_ fullerene, which was found to be 5 g/kg both for oral or intraperitoneal administration to rats [13]. No toxic effects or death have been fixed under the action of C_60_ fullerenes after their oral administration to rats in total dosage of 2 g/kg for 14 days [24]. Finally, it was shown [13] that water-soluble C_60_ fullerenes administered intraperitoneally to rats (0.5 mg/kg) were subjected to clearance from the organism within 2–4 days.

The animals in groups 1 and 2 were anaesthetized with ketamine (100 mg/kg “Pfizer”, USA) combined with xylazine (10 mg/kg, “Interchemie”, Holland), tracheostomized and artificially ventilated (out of necessity). The left and right *TS* muscles were separated from the surrounding tissue, and their tendons were detached at the distal insertions. The *n. tibialis* was separated from the tissue and cut proximally, and all branches of the nerve, except those innervating the *TS*, were cut. This nerve was mounted on a bipolar platinum wire electrode for electrical stimulation. The hindlimb muscles and nerves were covered with paraffin oil in a pool formed from skin flaps. The *TS* muscle was connected via the Achilles tendon to the servo-control muscle puller. The muscle tension was measured by semi-conductor strain gauge resistors glued on a stiff steel beam mounted on the moving part of a linear motor.

**Fig. 2:**
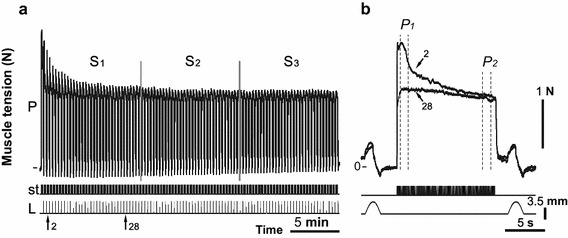
Strength of the contralateral (*right*) *m. triceps surae* (*TS*) contraction during the 2nd series of 30-min intermittent stimulations of the *n. tibialis* at 42 min after C_60_FAS administration into the left *TS* (**a**). The superposition of individual tetanic contractions 2 and 28 are presented at the higher time scale (**b**). *P* muscle force, *st* stimulation mark, *L* muscle length (mm); *S*_*1*_ *, S*_*2*_ *and S*_*3*_ equal parts of the stimulus series, used for the quantitative analysis of data; *P*_*1*_ and *P*_*2*_ sites at the beginning and at the end of tetanic contraction

To induce muscle fatigue, 1–3 (30 min duration) series intermittent high-frequency electrical stimulation was used (Fig. 2a), separated by rest intervals of 10–20 min. Each series consisted of trains of 0.2-ms rectangular pulses at a rate of 40/s at 12.4 s duration and separated by 5 s intervals of rest (Fig. 2b). The stimulus current was set to 1.3–1.4 times the motor threshold. Note, if muscle fatigue developed in less than 30 min, stimulation was interrupted (it was predicted that fatigue development occurred when there was a muscle force decrease of more than 50% of the initial data). After the end of the 12.4-s-stimulation, the muscle was stretched, and the change in length had a bell-shaped form (one period of 4 Hz sinusoidal signal with corresponding phase locking) of 3.5 mm amplitude and 2 s duration (Fig. 2b; bottom row). The muscle reaction to the stretches appeared as a tension increase after continuous stimulation. These stretches were applied before the post-stimulation twitches to remove, or at least diminish, the after-effects remaining from the continuous stimulation [1]. The signals (stimulus pulses, muscle tension and other) were sampled via DAC-ADC device (CED Power 1401).

### Biochemical experiment

For biochemical analysis, the excised *m. triceps surae* (soleus and gastrocnemius) were rapidly dissected, free of fat and tendon, divided into several portions and stored in liquid N_2_. For reduced glutathione (*GSH*) analysis, tissue samples were transferred into a medium containing 1 N perchloric acid (1:10 w/v) and homogenized with a motor-driven Potter–Elvehjem glass homogenizer. The resultant homogenate was centrifuged at 10,000*g* for 10 min (4 °C). The *GSH* content was spectrophotometrically measured [25]. For the enzyme activity assays and *H*_*2*_ *O*_*2*_ and lipid peroxidation assays, the muscle samples were thawed and homogenized in 50 mM phosphate buffer with 2 mM EDTA (pH 7.4) at 4 °C (1:9 w/v). Homogenates were then centrifuged for 15 min at 15,000*g* (4 °C), and the post mitochondrial supernatant was stored at −70 °C.

Oxidative damage in the tissue was measured using the thiobarbituric acid reactive substances (*TBARS*) assay. *TBARS* were isolated by boiling tissue homogenates for 15 min at 100 °C with thiobarbituric acid reagent (0.5% 2-thiobarbituric acid/10% trichloroacetic acid/0.63 M/dm^3^ hydrochloric acid) and measuring the absorbance at 532 nm. The results are expressed as nM *TBARS*/mg protein, using ɛ = 1.56 × 10^5^ dm^3^/M^1^/cm^1^ [26].

The *H*_*2*_ *O*_*2*_ concentration in the tissue homogenates was measured using the FOX method, which is based on the peroxide-mediated oxidation of Fe^2+^, followed by the reaction of Fe^3+^ with xylenol orange (o-cresolsulphonephthalein 3′,3″-bis[methylimino] diacetic acid, sodium salt). This method is extremely sensitive and is used to measure low levels of water-soluble hydroperoxide present in the aqueous phase. To determine the *H*_*2*_ *O*_*2*_ concentration, 500 μl of the incubation medium was added to 500 μl of assay reagent (500 μM ammonium ferrous sulphate, 50 mM H_2_SO_4_, 200 μM xylenol orange, and 200 mM sorbitol). The absorbance of the Fe^3+^-xylenol orange complex (A_560_) was detected after 45 min. Standard curves of *H*_*2*_ *O*_*2*_ were obtained for each independent experiment by adding variable amounts of *H*_*2*_ *O*_*2*_ to 500 μl of basal medium mixed with 500 μl of assay reagent. Data were normalized and expressed as μM *H*_*2*_ *O*_*2*_ per mg protein [27].

Catalase activity was measured by the decomposition of hydrogen peroxide, determined by a decrease in the absorbance at 240 nm [28].

*GSH* was determined using Ellman’s reagent. One millilitre of supernatant was treated with 0.5 ml of Ellman’s reagent (5.5′-dithio-bis-nitrobenzoic acid in abs. ethanol) and 0.4 M Tris HCl buffer with 2 mM EDTA, pH 8.9. The absorbance was read at 412 nm in a spectrophotometer [25].

The protein concentration was estimated using the method of Bradford with bovine serum albumin as a standard. All chemicals were purchased from Sigma, Fluka and Merck and were of the highest purity.

### Data analysis

In the electrophysiological part of the study, each stimulation series (30 min) was divided into three equal portions (Fig. 2a), which were averaged (maximum 33 stimulation in one portion). The average value of the first portion was set to 100%, and the other series were normalized in relation to this (for each hindlimb). The peak amplitudes of the front (*P*_*1*_) and rear of the front (*P*_*2*_) (maxima amplitudes at the site, duration of 1 s, Fig. 2b) of the muscle strength of each single series (12.4 s) were identified and the difference between *P*_*1*_ and *P*_*2*_ (Δ*P*) was calculated. This difference determines the dynamic component of the muscle force decrease in a short period of continuous stimulation. Mean values (mean ± SD) of the *TS* muscle strength before and after F-injection were compared using a two-way statistical analysis of variance (ANOVA). The factors of variation included two conditions, time and the effects of the C_60_FAS. A Bonferroni post hoc analysis was used to determine the differences between groups. The level of significance was set at p < 0.001.

Biochemical data are expressed as the means ± SEM for each group. The differences among experimental groups were detected by one-way ANOVA followed by Bonferroni’s multiple comparison test. Values of p < 0.05 were considered significant.

## Results

### Analysis of AFM and SANS data

Because the C_60_ fullerene particle size directly correlates with their biodistribution and toxicity [29, 30], the AFM and SANS studies were performed.

The AFM images (Fig. 1a, b) clearly demonstrate randomly arranged, individual C_60_ fullerenes (0.7 nm in diameter) and their bulk clusters with a height of 1.5–200 nm. At the same time, some individual C_60_ fullerene aggregates with a height of >200 nm are also seen in the AFM image (Fig. 1b). The results obtained are consistent with the theoretical calculations and experimental measurements [20, 21, 31, 32] and demonstrate the polydispersity of the C_60_FAS used in our study.

Experimental SANS curve for C_60_FAS is shown in Fig. 1c. The scattering curve of C_60_FAS is well described by the form-factor of polydisperse spherical particles. The mean radius of gyration of the particle cross section, R_g_, and pair distance distribution function, P(r), were found by using indirect Fourier transformation (IFT) approach [33]. We can calculate the radius of particles, R, present in the C_60_FAS according to well-known equation R_g_^2^ = 0.6R^2^ assuming of homogeneous and spherical of C_60_ fullerene clusters. This conclusion follows from previous experimental data [20, 21] and the estimates of the average cluster density according to the contrast-variation experiments [31, 32, 34]. The data given by this procedure indicate that C_60_FAS consists of C_60_ fullerene sphere-like nanoparticles with an average size of ~56 nm that is in a good agreement with above AFM data.

It is known [35, 36] that the permeability and cytochemical behavior of nanoparticles strongly depend on their size and, correspondingly, mass (number) distribution. In this regard, our previous studies [16, 18, 19, 29] clear demonstrate that the used C_60_ fullerene nanoparticles can effectively penetrate through the plasma membrane of cells by passive diffusion or endocytosis (depending on the size) and do not exhibit cytotoxic effects.

### Electrophysiological experiments

**Fig. 3:**
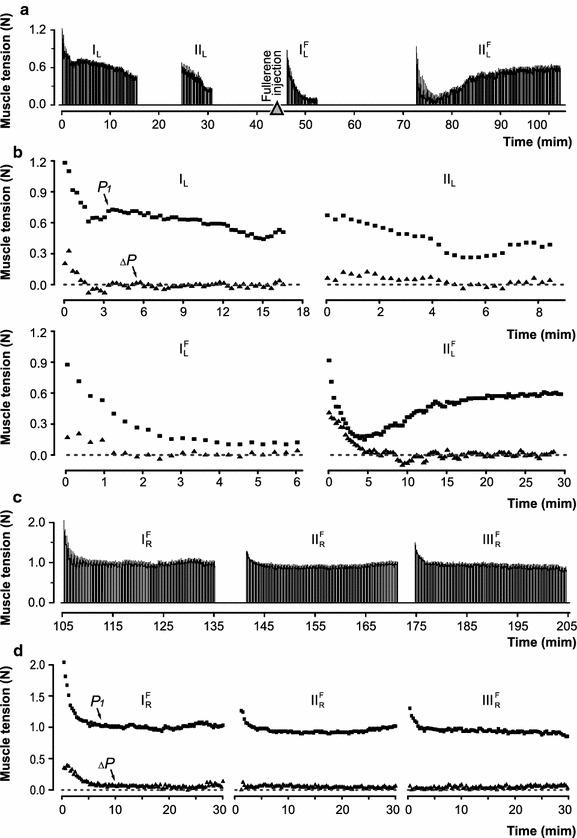
Strength of the ipsi- and contralateral *m. triceps surae* (*TS*) contraction induced by electrical fatigue stimulation before and after C_60_FAS injection into the *left TS*: **a**,** c** time protocol registrations of the *left* and *right TS* contraction, respectively (*triangles* indicate the moment the of the C_60_FAS injection); **b**,** d **amplitude values of the muscle force (*P*_*1*_) at the beginning of single tetanic contractions (*squares*) and Δ*P* (the difference between the force values at the beginning and at the end of muscle contraction; *triangles*). The rapid development of fatigue (**a**) (decrease in the muscle strength of more than 50%) led to shortening of the stimulation time (I–III). Designations for I–IV (**a**) and I–III at (**c**) correspond to recordings on (**b**) and (**d**), respectively. Indices: *L, R* left and right *TS*; *F* registration of muscle force after the administration of C_60_FAS into the left *TS*

Changes in the *TS* force reaction under fatigue conditions due to prolonged high frequency stimulation (30 min, 40/s) of the *n. tibialis* for animal groups 1 (before and after administration of saline solution) and 2 (before the application of C_60_FAS) did not significantly differ. The analysis was performed by determining the force level at the beginning (*P*_*1*_) and end (*P*_*2*_) of single tetanic contractions and the difference between these values (Δ*P*), which determines the dynamic component of the force decrease during a short period of continuous stimulation (Fig. 2b). The muscle was considered tired if the amplitude of the single tetanic contractions decreased by more than 50% relative to the initial level. When muscle fatigue was reached, the stimulation was stopped and followed by a 10–20 min rest period. Therefore, in the case of one animal, as a result of muscle fatigue stimulation of the left *TS*, a 50% fatigue level was reached in approximately 12 min; during the next 4 min of stimulation, it continued to decrease [Fig. 3a (I_L_), b(I_L_)]. After 10 min of rest, a single tetanic contraction force was slightly restored, but it did not reach the initial muscle activity level and continued to decrease rapidly [Fig. 3a (II_L_), b(II_L_)]. In this case, there was also a simultaneous decrease in the dynamic component of the force drop Δ*P*. Note that the dynamic component was the most highly expressed at the beginning of the first experimental series and that the *P*_*1*_ amplitude was higher relative to the *P*_*2*_ amplitude [Fig. 3b (II_L_)]. After tetanic contractions for 1.5–2 min, difference between amplitudes *P*_*1*_ and *P*_*2*_ was reduced to zero, with moderate variations both in one and the opposite direction over the additional fatigue stimulation period. Simultaneously with the decrease in Δ*P* values, there was a constant decrease in the developed force. In the following stimulation series, after a period of rest, the initial amplitude of the dynamic component was usually decreased [Fig. 3b (II_L_, III_L_)].

When a predetermined level of muscle fatigue was reached, C_60_FAS (0.1–0.15 mg/kg) was injected intramuscularly [at 45 min after the beginning of fatigue stimulation; Fig. 3a $({\text{I}}_{{ {\text{L}}}}^{\text{F}}$, ${\text{II}}_{{ {\text{L}}}}^{\text{F}}$), b(${\text{I}}_{{ {\text{L}}}}^{\text{F}}$, ${\text{II}}_{{ {\text{L}}}}^{\text{F}}$)]. At the same time, the dynamic changes in the muscle strength level in response to stimulation reflected the further development of fatigue, and the single contraction forces were reduced rapidly [Fig. 3b(${\text{I}}_{{ {\text{L}}}}^{\text{F}}$)]. However, F-injection led to the gradual recovery of the isometric force levels (at 32 min after drug application; Fig. 3a [(${\text{II}}_{{ {\text{L}}}}^{\text{F}}$), b $({\text{II}}_{{ {\text{L}}}}^{\text{F}}$)]. The appearance of negative Δ*P* values (*P*_*2*_ amplitude increase compared to *P*_*1*_ amplitude) indicated the beginning of the recovery [Fig. 3b (${\text{II}}_{{ {\text{L}}}}^{\text{F}}$), 10th min]. In this series of stimulations, the level of the muscle contraction force was recovered to that developed during the initial stages of fatigue stimulation.

Power reaction of the right *TS* was significantly different from the left *TS*. Notably, the *TS* of the right limb was not previously fatigued before the F-injection (Fig. 3c, d). At 52 min after drug administration, a certain force muscle decrease was observed. In this case, the *P*_*1*_ amplitude was higher than the *P*_*2*_ amplitude, as indicated by the increase in Δ*P* values [Fig. 3c (${\text{I}}_{{ {\text{R}}}}^{\text{F}}$), d (${\text{I}}_{{ {\text{R}}}}^{\text{F}}$)]. However, at 6 min after the beginning of fatigue stimulation, the force developed by the muscle appeared at a certain stationary level, which was held during the experimental series. The difference between the *P*_*1*_ and *P*_*2*_ amplitudes disappeared (value of Δ*P* decreased to zero), which may indicate a constant force level at the time of loading. It is significant that the muscle maintained the developed force level for an additional 1 h [Fig. 3c (${\text{II}}_{{ {\text{R}}}}^{\text{F}}$, ${\text{III}}_{{ {\text{R}}}}^{\text{F}}$), d(${\text{II}}_{{ {\text{R}}}}^{\text{F}}$, ${\text{III}}_{{ {\text{R}}}}^{\text{F}}$)]. For this muscle, the total time of the decrease of the isometric force contraction by 50% was 120 min after drug administration. For comparison, the control duration of the fatigue occurrence period was 42 min.

**Fig. 4:**
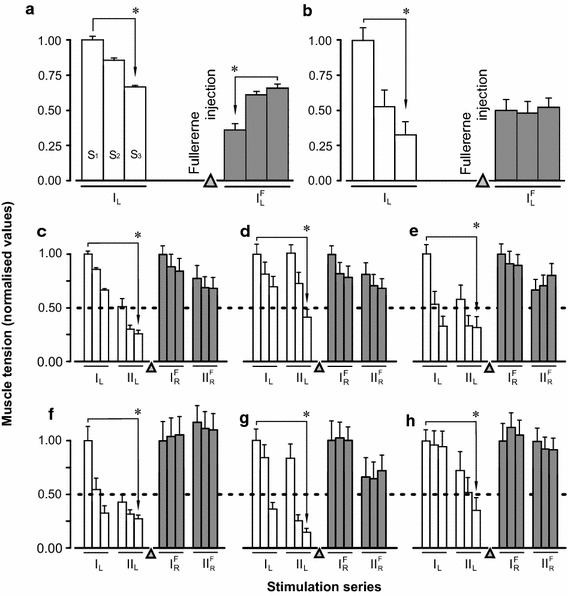
Averaged characteristics (mean ± SD) of normalised (to average values S1) values of the muscle strength during different parts of the fatigue stimulation (S1, S2, and S3; Fig. 2) before and after (*white* and *grey bars*, respectively) C_60_FAS administration into the left *m. triceps surae* (*TS*): **a**,** b** the results of two fatigue tests before and after C_60_FAS administration into the left *TS*; **c**–**h** the results of six fatigue tests of the left *TS* (*open bars*) before C_60_FAS administration, and right *TS* (*grey bars*) at 52 min after C_60_FAS administration into the left *TS*. *Asterisks* significant differences (p < 0.001) between the muscle strength during time intervals S_1_ and S_3_ in one or more series of the stimulation. *I, II* successive series of fatigue stimulations (normalisation performed by S_1_ in series I). Indices: *L, R* left and right *TS*; *F* registration of muscle force after the administration of C_60_FAS into the left *TS*. *Triangle marks* the moment of C_60_FAS injection

In Fig. 4a, b, a comparison of the force level changes developed by the left *TS* before (I_L_) and after (${\text{I}}_{{ {\text{L}}}}^{\text{F}}$) F-injection in two different experiments is presented. The statistical analysis showed a significant (p < 0.001) force decrease during the series of fatigue stimulation before C_60_FAS administration [Fig. 4a (I_L_), b(I_L_)]. After F-injection, a recovery of the muscle force for the first animal [Fig. 4a (${\text{I}}_{{ {\text{L}}}}^{\text{F}}$)] and its holding for the second animal [Fig. 4b (${\text{I}}_{{ {\text{L}}}}^{\text{F}}$)] was observed. At the end of the experimental series, the recovery of the active muscle force response was significant compared to that at the beginning and nearly reached the control values. The analysis showed a significant effect for the factors: drug administration (D) and time (T) after administration and their interaction. The corresponding results of this analysis were as follows: F(D) = 2904.47, p(D) < 0.001, F(T) = 42.420, p(T) < 0.001, F(DxT) = 1350.58, p(DxT) < 0.001 (first animal) and F(D) = 122.80, p(D) < 0.001, F(T) = 1058.29, p(T) < 0.001, p(DxT) = 1287.35, p(DxT) < 0.001 (second animal). The data obtained in all experiments (Fig. 4c–h) indicate that the decrease in the developed force after C_60_FAS administration (${\text{I}}_{{ {\text{L}}}}^{\text{F}}$, ${\text{II}}_{{ {\text{R}}}}^{\text{F}}$) was almost two times slower than in controls. The maximum significant reduction of the muscle force developed during the entire period of fatigue stimulation was 44% after drug administration, whereas in the control this was 85%. For all experimental animals, similar dynamics of the force level decrease in the control and its more gradual decrease after F-injection were observed.

### Biochemical experiments

**Fig. 5:**
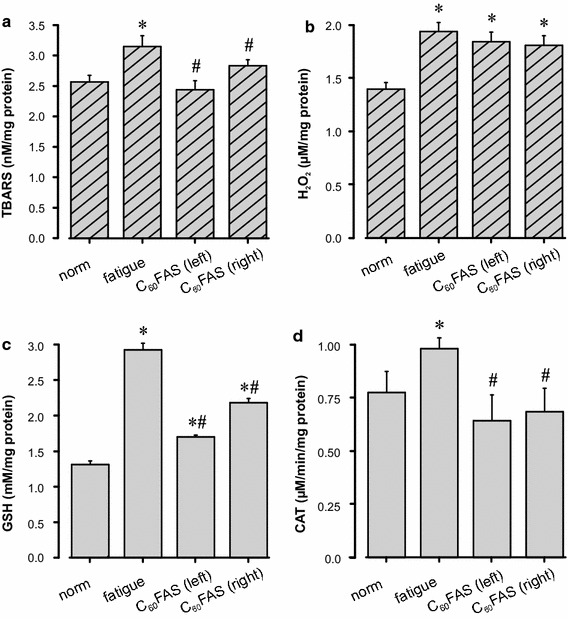
Indicators of the prooxidant-antioxidant balance in the *m. triceps surae* (*TS*) of rats. The concentration of thiobarbituric acid reactive substances (*TBARS*) (**a**), hydrogen peroxide (*H*_*2*_ *O*_*2*_) (**b**), glutathione (*GSH*) (**c**) and catalase (*CAT*) (**d**) are in intact animal muscles (norm), with the left fatigued TS (fatigue) and after C_60_FAS administration ipsi- and contralaterally [C_60_FAS (*left*) and C_60_FAS (*right*), correspondingly]. Values are the mean ± SEM, n = 6. *p < 0.05 vs. “norm”; ^#^p < 0.05 vs. “fatigue”

During long-term stimulation of the muscle, metabolic processes change and are a main factor of muscle fatigue. As a result of the fatigue test, the accumulation of lipid peroxidation secondary products and changes in the levels of antioxidants in the tissue of the fatigued muscle were determined. The data clearly demonstrate the increased level of peroxidation and oxidative stress marker *TBARS* and *H*_*2*_ *O*_*2*_ after fatigue stimulation (Fig. 5a, b). This increase was significant in relation to the intact muscle (*‘norm’*) and was 23% (p < 0.05) for *TBARS* and 38% (p < 0.05) for *H*_*2*_ *O*_*2*_. After C_60_FAS administration into the left *TS*, the *TBARS* concentration was significantly reduced compared to fatigue as follows: 29% (p < 0.05) for the left *TS* and 12% (p < 0.05) for the right one. The *H*_*2*_ *O*_*2*_ level decreases in comparison to the ‘*fatigue*’ group (by 6% for the left *TS* and 7% for the right one), although the *H*_*2*_ *O*_*2*_ level remained higher in relation to the intact group (p < 0.05). In turn, in response to such changes in the working muscle, an activation of endogenous antioxidants occurred. During fatigue stimulation, the amount of muscle *GSH* quantitatively increased more than two-fold (p < 0.05) and the activity of the antiperoxide enzyme *CAT* also increased. After C_60_FAS administration, the *GSH* and *CAT* activities were significantly decreased compared to the group *‘fatigue’* by 41.8 and 15.4% for *GSH* and 53 and 43% for *CAT* (p < 0.05) for the left and right *TS*, respectively (Fig. 5c, d).

## Discussion

In this study, we investigated changes in the contraction force of the rat *m*. *triceps surae* under fatigue development before and after C_60_FAS administration. We did not use a level of stimulation above 40 Hz, and the rest period between the experimental series was 15–20 min [2]. This experimental approach allows us to analyse the nature of the muscle contraction force parameter changes under fatigue stimulation before C_60_FAS application (into the left *TS*) and directly after F-injection. A marked decrease in the muscle effort level before C_60_FAS administration (control) was observe in the all experiments both I_L_ and II_L_ stimulation series (Fig. 4a–h). It was the result of modified stimulation pattern action, which was due to the influence of the central and peripheral mechanisms of the development of skeletal muscle fatigue [2]. After intramuscular injection of the C_60_FAS partial ipsilateral *TS* muscle recovery was registered in two rats. However, the main finding was observed after the application of C_60_FAS. Not significant a slower effort decrease, followed by the prolonged retention of a certain level was recorded contralaterally in all animals. Decrease in the muscle contraction force was developed more slowly after C_60_FAS administration compared to the control. It indicates a deceleration of the fatigue process, and the strength restraint at the constant level for a long time (120 min) indicates an increase in the muscle endurance during such conditions. The data obtained in this study indicate that after drug injection, the time for the *TS* force maximal level decrease to 44% was 120 min. At the same time in the control, the force level of this muscle during the same period decreased to 85%. We suppose, it was caused by antioxidant effects C_60_FAS on the fatiguing muscle. The duration of the muscle recovery and its rest periods are also important factors for maintaining efficiency and the normal physiological state of the muscle during dynamic work execution [12]. The dynamic component of the single tetanic contraction is likely a reflection of the interaction of the efficiency of the initial increase of the fast motor unit contractile properties and processes of the fatigue strength reduction [37]. Thus, recovery of muscle strength after F-injection both for the preliminary tired and at fresh muscles indicate, that water-soluble pristine C_60_ fullerenes can penetrate through the plasma membrane of cells [18, 19] and render of powerful antioxidant effect on the contractile apparatus of striated muscle, thereby facilitating its functional recovery after experimentally induced fatigue.

Under a moderate external load on the muscle, metabolism occurs aerobically. In the actively contracted muscle, metabolism significantly increases, resulting in the accumulation of secondary oxidation products in muscle fibres, which leads to fatigue development [38]. These metabolic processes are a source of oxygen free radicals and contribute to the intensification of lipid peroxidation processes [39–41]. The presence of such metabolism products prevents the adequate implementation of muscle work and increases the duration of the recovery period. Strenuous exercise and endurance training cause oxidative stress in skeletal muscle and can therefore alter the prooxidant-antioxidant balance [42, 43]. Despite extensive research over the years, the relationship between free radical generation, antioxidant enzymes and exercise in skeletal muscle remains controversial [44, 45]. These discrepancies may be related to differences in exercise mode, intensity, duration of the training program, and muscle fibre type. Skeletal muscles are highly heterogeneous. Each muscle fibre type has distinct metabolic characteristics and oxidative potential as well as antioxidant defence capacity [41]. In our study, as a result of fatigue stimulation in working muscle, there was a significant increase in the secondary products of lipid peroxidation and *H*_*2*_ *O*_*2*_ compared to the intact (unstimulated muscle) muscle (Fig. 5). During intense (physical activity) contraction, the flow of oxygen through muscle cells is greatly increased. High levels of oxygen uptake (up to 100-fold) can lead to excessive ROS generation and are implicated in fatigue, muscle soreness, and myofibril disruption [45]. Moreover, another potential mechanism involved in the oxidative stress response to high-intensity exercise is the redistribution of blood flow, such as elevated blood flow in the heart, lung, and red slow-twitch muscle fibres, leading to increased mitochondrial respiration, which results in an increase in the production of ROS. We found that long-term electrical stimulation of the muscle induced a significant increase in *TBARS* and *H*_*2*_ *O*_*2*_ content that led to an increase of *CAT* activity and *GSH* content in both fast- and slow-twitch muscle fibres. In this case, after C_60_FAS administration, the oxygen metabolite concentration was significantly lower. This confirms the previous data regarding the protective effect of C_60_FAS on the immune and antioxidant systems of the body in various pathologies [15, 46]. The mechanisms of effects of this drug can positively influence the processes of endurance and recovery of the active muscles, inactivating the products of its metabolism.

Increased amounts of *GSH* in the stimulated muscle (without drug administration and after its application) are evidence of the compensatory activation of the endogenous antioxidant systems on the irritant action of sufficient strength (Fig. 5). Many studies showed that during intense stress, there is a significant decrease of reduced *GSH* and an increased concentration of its oxidative form in the myocardium and *m. soleus* [47, 48]. Simultaneously, contradictory data were obtained in the experiments studying endurance [47, 49]. It was found that under physical activity, the amount of reduced *GSH* in the *m. gastrocnemius* and *DVL* increase. It is likely that in *m. soleus*, a muscle with a high content of myoglobin, all metabolic and biochemical processes occur under aerobic conditions, which use a large number of mitochondrial enzymes, and the accumulation of oxidized *GSSG* does not have time to reduce [50]. At the same time, the above mentioned processes in the *m. gastrocnemius* occur anaerobically, in contrast to the *m. soleus*. This causes a slow oxidation process and increases the amount of reduced *GSH* [51, 52]. Under fatigue, after C_60_FAS administration, the *GSH* content was somewhat reduced compared to the “fatigue” state, indicating a reduction in oxidative stress and a normalization of the pro- and antioxidant balance in rat muscle tissue (Fig. 5).

An increase of *H*_*2*_ *O*_*2*_ during exertion leads to an increase in *CAT* enzyme activity that has a protective antioxidant function by catalysing the decomposition of hydrogen peroxide to water and oxygen. These results are confirmed by previously obtained data from acute experiments on rats with *DVL* stimulation [47, 52]. An increase of the enzyme activity in response to exercise was also shown in humans [53]. Moreover, some studies indicate an absence of any changes in *CAT* concentration in the muscles during physical activity [44, 54, 55]. In fact, several reports demonstrated decreases in catalase activity in both oxidative and mixed fibre limb muscles [56, 57]. In our study, after C_60_FAS administration under fatigue development, the *CAT* activity was significantly reduced compared to pure fatigue and remained at the control level. It is hypothesized that C_60_FAS influence the content and activity of endogenous antioxidants and prevent the occurrence of fatigue in actively contracting muscle, thereby contributing to maintenance of its normal physiological state.

Free radical processes increasing is the main pathogenic factor during skeletal muscles fatigue development [58]. Under significant physical activity there is highly overproduction of free radicals in muscle tissue that intensifies the processes of lipid peroxidation, cell membranes damage and antioxidant enzymes inactivation [59]. The active oxygen metabolites cause direct inhibition of respiratory chain mitochondrial enzymes and reducing the balance of ATP/ADF [59]. The above processes in the background of the lactate accumulation with subsequent development of acidosis and blockage of membrane Ca^2+^ channels lead to a pronounced energy deficit and a significant functional activity reduction of muscle tissue [60].

It is known that application of different nature exogenous antioxidants leads to a significant reduction of fatigue skeletal muscle during intense physical activity and increases the onset time of muscle fatigue under prolonged intense endurance exercise [10, 61, 62]. These data demonstrate the feasibility of using antioxidants to correct the level of oxidative stress in the muscle tissue under extreme influences on the body and its efficiency increasing. Since pristine C_60_ fullerenes, as previously shown in various models in vitro and in vivo [13, 15, 63], actively bind free radicals and display a powerful antioxidant properties of direct action, we can assume that the application of water-soluble C_60_ fullerenes led to the prooxidant-antioxidant balance normalization in the muscle tissue of rats and helped improve the dynamic parameters of muscle contraction.

## Conclusion

The use of C_60_FAS, even at a low therapeutic dose (0.1–0.15 mg/kg) leads to a reduction in the recovery time of the muscle contraction force (after its complete exhaustion state) on the one hand, and an increase in the time of the muscle active work (endurance) until fatigue development on the other. This result illustrates the effect of C_60_FAS, along with other possible mechanisms, on prooxidant-antioxidant homeostasis in the muscle tissue of rats.

## Abbreviations

C_60_FAS: pristine C_60_ fullerene aqueous colloid solution
*H*_*2*_*O*_*2*_: hydrogen peroxide
*TBARS*: thiobarbituric acid reactive substances
*GSH*: reduced glutathione
*CAT*: catalase
ROS: reactive oxygen species
NAD^+^: nicotinamide adenine dinucleotide
HCl: hydrochloric acid
AFM: atomic force microscopy
SANS: small-angle neutron scattering
FOX: ferrous ion oxidation xylenol orange
H_2_SO_4_: sulphuric acid
EDTA: ethylenediaminetetraacetic acid
DAC: digital to analogue converter
ADC: analogue to digital converter
ANOVA: analysis of variance
DVL: deep portion of vastus lateralis muscle
GSSG: glutathione disulfide
